# Constructing a highly efficient multifunctional carbon quantum dot platform for the treatment of infectious wounds

**DOI:** 10.1093/rb/rbae105

**Published:** 2024-08-24

**Authors:** Hangzhen Zhang, Jiafan Bai, Xiangli Chen, Linyu Wang, Wenzhen Peng, Yuancong Zhao, Jie Weng, Wei Zhi, Jianxin Wang, Kai Zhang, Xingdong Zhang

**Affiliations:** Key Laboratory of Advance Technologies of Materials, Ministry of Education, College of Medicine and School of Materials Science and Engineering, Southwest Jiaotong University, Chengdu 610031, China; Key Laboratory of Advance Technologies of Materials, Ministry of Education, College of Medicine and School of Materials Science and Engineering, Southwest Jiaotong University, Chengdu 610031, China; Key Laboratory of Advance Technologies of Materials, Ministry of Education, College of Medicine and School of Materials Science and Engineering, Southwest Jiaotong University, Chengdu 610031, China; Key Laboratory of Advance Technologies of Materials, Ministry of Education, College of Medicine and School of Materials Science and Engineering, Southwest Jiaotong University, Chengdu 610031, China; Department of Biochemistry and Molecular Biology, College of Basic and Forensic Medicine, Sichuan University, Chengdu 610041, China; Key Laboratory of Advance Technologies of Materials, Ministry of Education, College of Medicine and School of Materials Science and Engineering, Southwest Jiaotong University, Chengdu 610031, China; Key Laboratory of Advance Technologies of Materials, Ministry of Education, College of Medicine and School of Materials Science and Engineering, Southwest Jiaotong University, Chengdu 610031, China; Key Laboratory of Advance Technologies of Materials, Ministry of Education, College of Medicine and School of Materials Science and Engineering, Southwest Jiaotong University, Chengdu 610031, China; Key Laboratory of Advance Technologies of Materials, Ministry of Education, College of Medicine and School of Materials Science and Engineering, Southwest Jiaotong University, Chengdu 610031, China; National Engineering Research Center for Biomaterials, College of Biomedical Engineering, Sichuan University, Chengdu 610064, China; National Engineering Research Center for Biomaterials, College of Biomedical Engineering, Sichuan University, Chengdu 610064, China

**Keywords:** multifunctional fluorescent carbon dots, antibacterial, immunomodulation, bacterial labeling, promoting angiogenesis

## Abstract

Antibiotic resistance poses a huge threat to public health, which has increased the difficulty and transmission of disease treatment, as well as the burden and cost of medical institutions. In response to the current problems and challenges in inflammation control and treatment of bacterial infected wounds, inspired by antibacterial mechanisms based on active elements such as N, S, Cu and tannic acid (TA), a highly efficient multifunctional carbon quantum dot platform was proposed in this study and constructed through their special assembly in a solvothermal reaction system for the treatment of infected wounds. By introducing active elements such as N, S and Cu, this carbon quantum dot platform is endowed with antibacterial properties, while also achieving good angiogenesis promoting performance through the use of ion Cu. Meanwhile, the good antioxidant activity of TA (one of the precursors used) enables this platform to have better immunomodulatory performance *in vivo*. The research results on the treatment of bacterial infection models indicate that the multifunctional carbon quantum dots obtained can accelerate the healing of infected wounds by inhibiting bacterial infection, regulating immunoreaction, accelerating collagen deposition and promoting angiogenesis. This multifunctional carbon quantum dot platform shows good clinical application prospects in treating bacterial infected wounds. Additionally, the fluorescence characteristics of such carbon dots can be expected to realize visual therapy in the future.

## Graphical abstract

**Figure:**
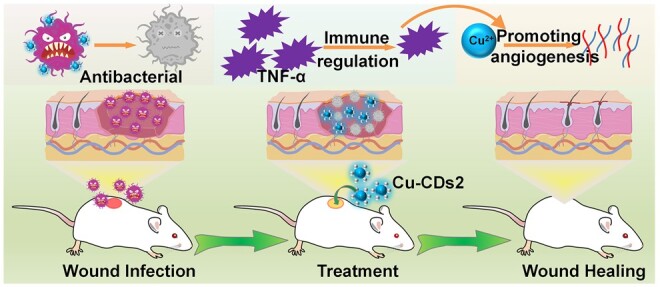

## Introduction

Bacterial infections have long been a major global public health challenge. The continued overuse and misuse of antibiotics has led to an increase in bacterial resistance. Therefore, the development of new antimicrobial agents has become more urgent [1, 2]. At present, researchers try to find a variety of methods to solve this issue, such as photothermal therapy, magnetic therapy, and the use of metals and metal-oxide nanoparticles [3–8]. However, these methods have some disadvantages in the treatment process that cannot be ignored. For example, photothermal therapy and magnetic therapy are limited in local treatment due to operational complexity and equipment requirements, while some metal and metal-oxide nanoparticles are both costly and toxic. Therefore, this needs to find a new strategy to develop a highly effective antibacterial agent with low toxicity, good biocompatibility and different from traditional small-molecule antibiotics. Carbon dots (CDs), a new type of nanomaterial, have attracted much attention in biomedical applications such as drug delivery [9], bioimaging [10] and antimicrobial agents [11] due to their unique physicochemical and optical properties [12]. They are one of the most widely studied antimicrobial materials with low drug resistance and good biocompatibility to date [13–15]. However, most reported antimicrobial CDs were endowed with antimicrobial function by means of coupling with certain antibacterial materials, such as antibiotics, peptides and quaternary ammonium salts, etc. or with the help of photodynamic power [16–19]. These methods have obvious drawbacks such as cumbersome preparation process, high cost and toxicity. Liu *et al.* [20] also reported a Cu-doped CD with nanoenzyme activity, which can achieve antibacterial effect through its enzymatic activity to generate reactive oxygen species. However, the related mechanism and system are complicated, making it difficult to be promoted.

**Figure 1.**
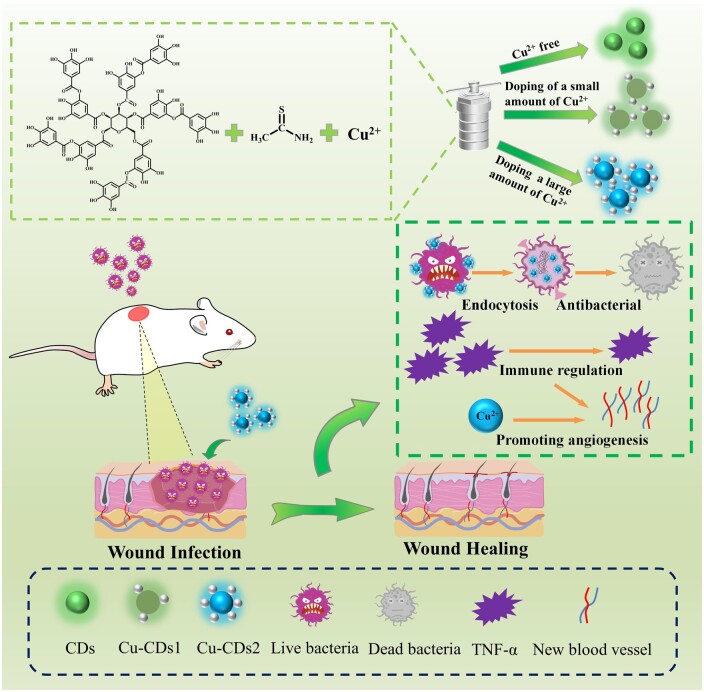
Schematic diagram of the synthesis of antibacterial CDs and their treatment in bacterial infections of wounds.

Tannic acid (TA), a naturally derived polyphenolic compound, has good biocompatibility, antioxidant, anti-inflammatory and *in vivo* immunomodulatory properties, as well as a certain degree of antimicrobial activity, which facilitate tissue repair [21–23]. As far as we know, there have been no reports on the synthesis of antibacterial CDs with TA as a precursor yet. In this study, TA would be used as a precursor to explore the synthesis of antibacterial CDs which would be expected to promote tissue repair, and its mechanism was also discussed. In view of the inability of a single TA to synthesize fluorescent CDs, we would functionalize the surface of the CDs by introducing some materials containing N and S heteroatoms to prepare CDs with strong antibacterial and antioxidant activities [24, 25]. In addition, considering that TA contains a large number of phenolic hydroxyl structures, they can provide a large number of reaction sites for the chelation of active metal ions (Cu^2+^, Zn^2+^, Mg^2+^, etc.), which is conducive to the loading of active metal ions and endows CDs with higher biological activity and more functions. Therefore, we designed a highly efficient synergistic antibacterial system by introducing Cu^2+^, which has antibacterial and vascularizing functions, into the material system.[26, 27] Compared with other antibacterial materials, the fluorescent CDs are smaller in size and easier to contact bacteria through multivalent interactions, thus disrupting the stability and permeability of the bacterial membrane. Also, it would be easier for them to reach every corner of the bacteria through endocytosis, thus affecting the metabolic process inside the bacteria, as well as the synthesis of DNA and protein, and ultimately inhibiting the proliferation of the bacteria, thereby achieving efficient antibacterial activity. In addition, the introduction of Cu^2+^ would effectively promote vascularization and then wound healing. This simple, green and low-cost synthesis of CDs that have good biocompatibility, *in vivo* immune regulation and high antibacterial efficiency will open up new ideas for the regeneration and repair of infected tissue (Figure 1).

## Results

### Morphological and structural characterization of carbon dots

**Figure 2.**
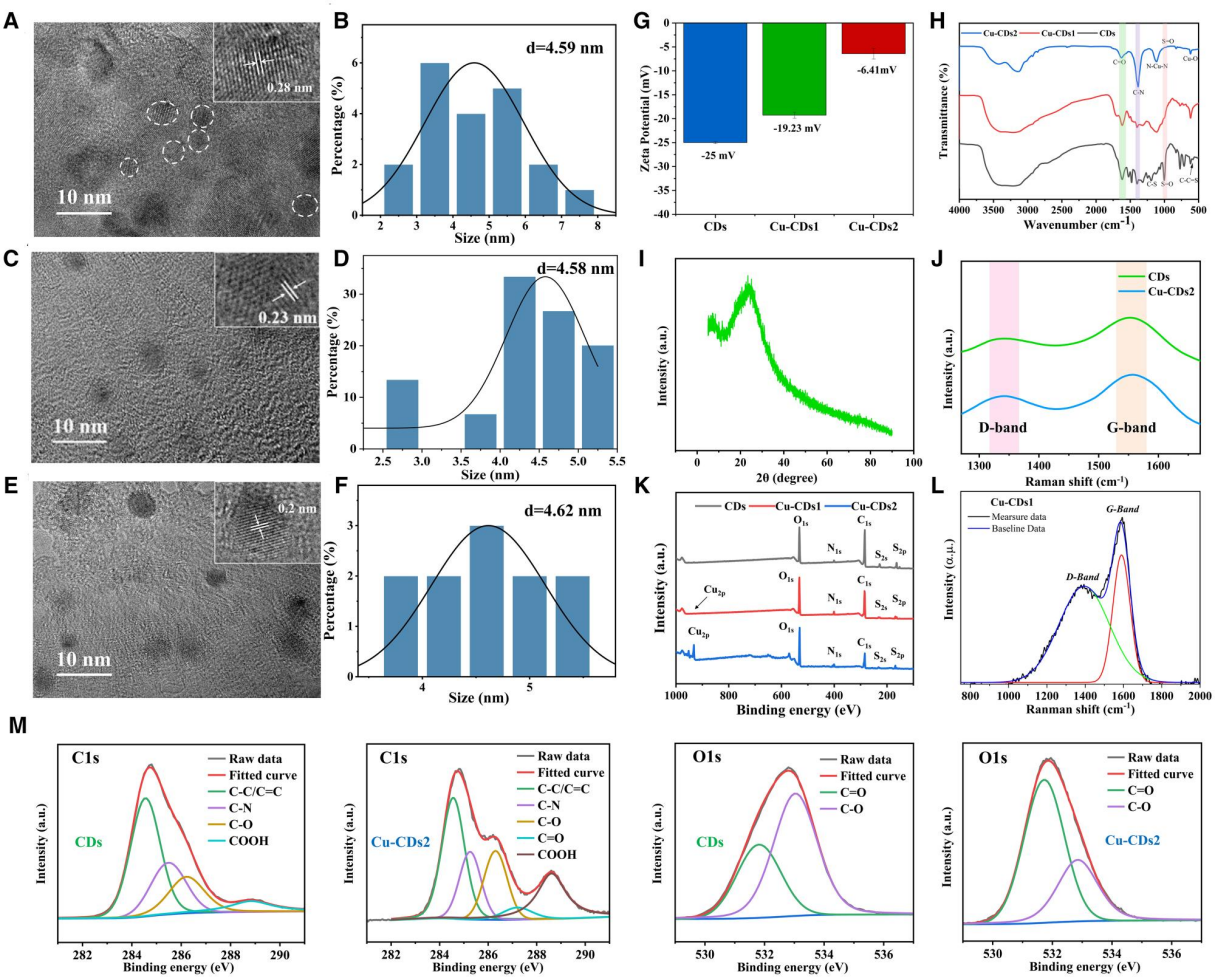
TEM and HRTEM images (inset) of (**A**) CDs, (**C**) Cu-CDs1 and (**E**) Cu-CDs2. Particle size distribution diagrams of (**B**) CDs, (**D**) Cu-CDs1 and (**F**) Cu-CDs2. (**I**) XRD spectra of CDs. (**J**) Raman spectra of CDs and Cu-CDs2. (**L**) Raman spectra of Cu-CDs1. (**G**) Zeta potential of CDs, Cu-CDs1 and Cu-CDs2. (**H**) FTIR spectra of CDs, Cu-CDs1 and Cu-CDs2. (**K**) The full XPS spectra of CDs, Cu-CDs1 and Cu-CDs2. (**M**) High-resolution XPS spectra of C 1s and O 1s of CDs and Cu-CDs2.

The morphologies of CDs, Cu-CDs1 and Cu-CDs2 were characterized using transmission electron microscopy (TEM), as shown in Figure 2A–F. It can be seen that CDs, Cu-CDs1 and Cu-CDs2 were well-dispersed with average particle sizes of about 4.59, 4.58 and 4.62 nm, respectively. High-resolution TEM (HRTEM) images show that both CDs, Cu-CDs1 and Cu-CDs2 had well-resolved lattice fringes with crystal plane spacing of 0.28, 0.23 and 0.2 nm, corresponding to the (020) and (100) crystal plane of graphitic carbon, respectively. X-ray diffraction (XRD) spectrum of CDs (Figure 2I) exhibits a strong diffraction peak around 24°, which is due to the typical vibration of graphitic carbon, indicating high crystallinity of CDs, thus being in good agreement with the TEM results. The Raman spectrum (Figure 2J) showed the degree of CDs defects and graphitization, both CDs and Cu-CDs2 showed two characteristic peaks at 1342 and 1554 cm^−1^, while Cu-CDs1 indicated two characteristic peaks at 1368 and 1593 cm^−1^, these peaks correspond to the disordered structure or defects (D band) and sp^2^ graphitic structure (G band) of the carbon material, respectively, showing that the carbon atoms are hybridized by disordered and sp^2^ hybridization [28, 29]. The intensity ratios of the D and G bands (*I*_D_/*I*_G_) were 0.85, 0.76 and 0.73 for CDs, Cu-CDs1 and Cu-CDs2, respectively, indicating that both the proportion of indeterminate carbon in the structure and the degree of graphitization increased from CDs to Cu-CDs2. Moreover, the zeta potentials of CDs, Cu-CDs1 and Cu-CDs2 were −25, −19.23 and −6.41 mV (Figure 2G), respectively, indicating that they had good dispersion properties and the surface potential of the material increased due to the doping of copper [30].

The compositions of the CDs and Cu-doped CDs were investigated using Fourier transform infrared (FTIR) spectra and X-ray photoelectron spectroscopy (XPS). As shown in Figure 2H, the FTIR spectra show that CDs, Cu-CDs1 and Cu-CDs2 possessed abundant polar functional groups, such as O-H/N-H (3500 − 3100 cm^−1^), C=O (1650 − 1600 cm^−1^), which ensured their good water solubility. Meanwhile, C=C (1530 − 1450 cm^−1^) and C-O (1200 − 1100 cm^−1^) were detected on the surface of CDs, while C-N (1410 − 1380 cm^−1^) was found on the surface of CDs, Cu-CDs1 and Cu-CDs2, indicating the formation of polyaromatic structures in the CDs during the synthesis process. Furthermore, some typical peaks of CDs were observed at 1190, 1063, 999 and 577 cm^−1^, which were attributed to the stretching vibration of C-S, S=O and C-C=S groups [31], respectively, indicating the successful doping of S on the surface of CDs. While the stretching vibration intensity of S=O at 999 cm^−1^ decreased and C=O at 1620 cm^−1^ increased for Cu-CDs1 and Cu-CDs, indicating that the phenolic hydroxyl group of TA underwent redox reactions with Cu^2+^ upon doping with copper ions [32], and two new strong absorption peak at 1090 and 622 cm^−1^ of Cu-CDs2 were attributed to the stretching vibration of N-Cu-N and Cu-O [33, 34], respectively, indicating that copper was incorporated during the synthesis of Cu doped CDs, resulting in successful formation of Cu coordination compound.

To further determine the element composition and valence state of the CDs, XPS were performed. As shown in Figure 2K, the full XPS spectra revealed that the surfaces of CDs, Cu-CDs1 and Cu-CDs2 contained five typical peaks of C 1s (284.2 eV), N 1s (400.9 eV), O 1s (532.1 eV), S 2s (228.1 eV) and S 2p (163.4 eV). It was further verified that N and S were involved in the formation process of CDs, which have affected their structure and properties, such as enhanced fluorescent properties and antibacterial activity. In particular, Cu-CDs1 and Cu-CDs2 also contained Cu 2p (932 eV) typical peaks, indicating the successful doping of Cu on the surface of Cu-CDs1 but with a low content of 0.13% (Supplementary Table S1). Therefore, in this study, the Cu-CDs2 with 5.65% Cu content was prepared by increasing the proportion of Cu^2+^ in the reactions system. In the high-resolution XPS spectrum (Figure 2M and Supplementary Figures S1 and S2), it can be seen that the C 1 s spectra can be deconvoluted into C-C/C=C (284.6 eV), C-N (285.5 eV), C-O (286.2 eV), C=O (287.2 eV) and COOH (288.8 eV). The N 1s spectra can be deconvoluted into pyrrole nitrogen (399.8 eV), graphite nitrogen (401.6 eV), and N-O (406.8 eV). The O 1s spectra can be deconvoluted into C=O (531.8 eV) and C-O (533 eV) [35, 36]. The S 2p spectra mainly consists of two peaks centered at 164 and 167.6 eV, which indicates the presence of sulfur in two forms, the former peak can be deconvoluted into S 2p^3/2^ (163.8 eV) and S 2p^1/2^ (165 eV), while the latter peak can be deconvoluted into peaks located at 168.1, 168.8 and 169.9 eV for C-SO_X_ (X = 2, 3, 4) [37]. The peaks at 932.8 and 934.9 eV in the Cu 2p spectra were corresponded to the Cu 2p^3/2^ feature of Cu^+^ and Cu^2+^, and the peaks at 952.4 and 954.5 eV were attributed to the Cu 2p^1/2^ feature of Cu^+^ and Cu^2+^ [20, 38]. The results further validated the successful introduction of N, S and Cu, which provided the structural basis for the antibacterial activity of N, S and Cu active elements and Cu^2+^ promoted vascularization. In addition, the increase of the COOH content in the C 1s spectra and C=O in the O 1s spectra (Supplementary Table S2) further demonstrated that the coordination and redox reactions between Cu^2+^ and the phenolic hydroxyl group of TA increased CDs’ oxidation degree, which is consistent with the analysis results of FTIR.

### Optical properties of carbon dots

**Figure 3.**
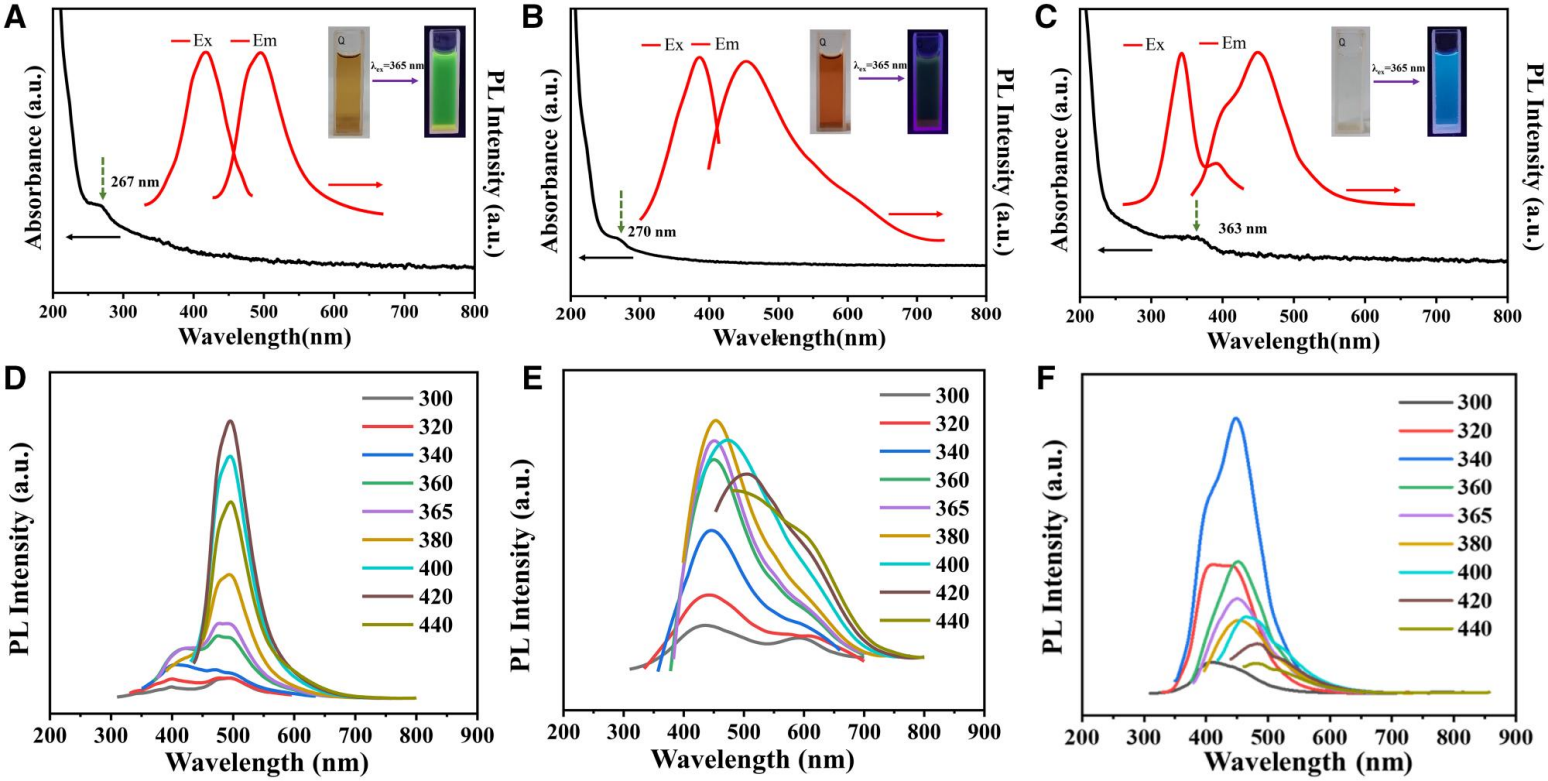
Absorption spectra, PL excitation and PL emission spectra (photographs of CDs under daylight (left) and UV light (excited at 365 nm) (right)) of (**A**) CDs, (**B**) Cu-CDs1 and (**C**) Cu-CDs2. PL emission spectra of (**D**) CDs, (**E**) Cu-CDs1 and (**F**) Cu-CDs2 under excitation of different wavelengths of light.

The UV-vis absorption spectra of CDs, Cu-CDs1 and Cu-CDs2 are shown in Figure 3A–C. Which exhibit an absorption peaks at 267 and 270 nm, respectively, corresponding to the π–π* transitions of aromatic sp^2^ domains in the carbon cores, while the absorption peak at 363 nm for Cu-CDs2 should be due to the *n*−π* transitions caused by edge transition of CDs. The edge band of CDs has been reported many times and refers to the edge atoms in the crystalline carbon nuclei, especially in some blue fluorescent CDs where this feature is often present [39]. As shown in Figure 3A, when CDs was dissolved in water, it appeared light yellow and exhibit bright green fluorescence under UV irradiation (*λ* = 365 nm). When a small amount of Cu^2+^ ions were doped in the raw material system, the prepared Cu-CDs1 showed a brownish yellow color when dissolved in water (Figure 3B), and they emitted dull green fluorescence under UV irradiation (*λ* = 365 nm), however, their fluorescence performance was relatively poor. Considering this, this study further doped more Cu^2+^ and allowed them to participate in the system reaction and prepared Cu-CDs2. As shown in Figure 3C, Cu-CDs2 showed a transparent and colorless state when dissolved in water, which was in sharp contrast to the color of the initial CDs solution (light yellow). Moreover, it exhibited blue fluorescence under 365 nm UV excitationn, and its fluorescence performance was improved compared to Cu-CDs1. Therefore, from the changes in solution color and fluorescence color, it is further verified that Cu^2+^ participated in the reaction in this system.

To further investigate the fluorescence properties of the prepared CDs, the analysis of fluorescence spectra were performed in this study. As shown in Figure 3D–F, the maximum emission peaks of CDs, Cu-CDs1 and Cu-CDs2 were at 495, 455 and 488 nm with quantum yields of 10%, 1% and 12%, respectively. We further tested the variation on the fluorescence properties of CDs, Cu-CDs1 and Cu-CDs2 at the same excitation wavelength. As shown in Supplementary Figure S3, the fluorescence emission wavelength was blue-shifted from CDs to Cu-CDs2, and the fluorescence intensity showed a decrease followed by an increase. The results indicated that the fluorescence performance of Cu-CDs1 was poor when doped with a small amount of Cu^2+^. While the Cu^2+^ content was increased, we obtained Cu-CDs2 with good fluorescence performance, which will provide the possibility for CDs and Cu-CDs2 to be used in fields such as fluorescent labeling. We further investigated the effects of different pH levels, NaCl concentration and hydrogen peroxide concentration environments, and laser radiation time on the fluorescence intensity of CDs and Cu-CDs2. Supplementary Figure S4 shows that the fluorescence intensity of CDs slightly decreased with increasing pH, but the relative change was not significant, while the fluorescence intensity of Cu-CDs2 remained basically stable with changing pH value. Even when CDs and Cu-CDs2 were in a medium environment of 2 mol/l NaCl, 0.1 mmol/l H_2_O_2_ or exposed to an Xe lamp for 1 h_,_ their fluorescence intensity still remained stable. The results indicate that CDs and Cu-CDs2 have good fluorescence stability, which provides a basis for their application in complex biological environments.

### *In vitro* antimicrobial test

**Figure 4.**
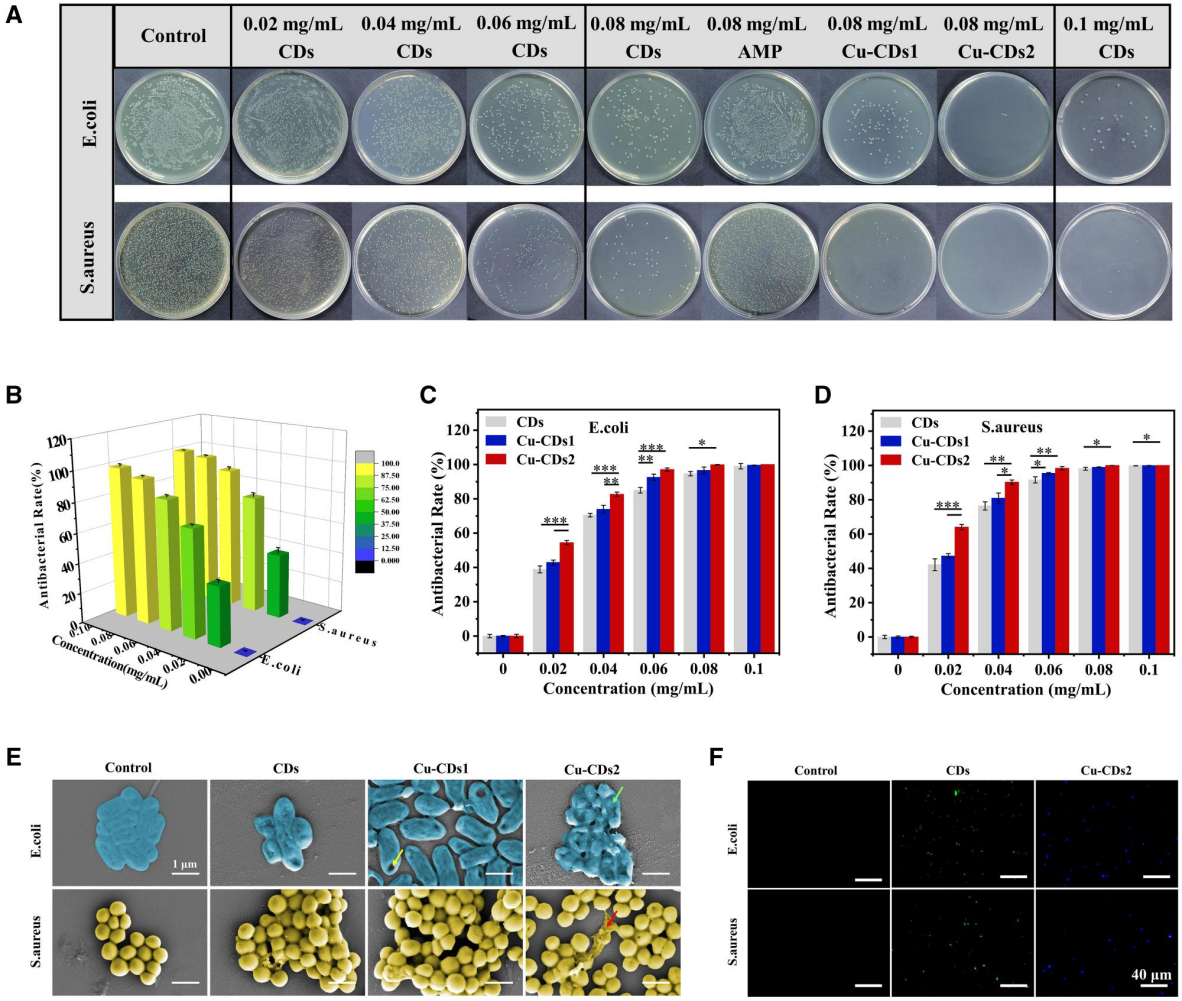
(**A**) Plate count images of *E. coli* and *S. aureus* after co-culture with different concentrations of CDs, AMP, Cu-CDs1 and Cu-CDs2. (**B**) Antibacterial rate 3D plots of CDs with different concentrations against *E. coli* and *S. aureus*. Antibacterial rate of CDs, Cu-CDs1 and Cu-CDs2 with different concentrations against (**C**) *E. coli* and (**D**) *S. aureus*. (**E**) SEM images of *E. coli* and *S. aureus* after treatment with CDs, Cu-CDs1 and Cu-CDs2 (The arrows point to perforation, collapse and outflow of bacterial contents, respectively). (**F**) Fluorescence microscopy images of *E. coli* and *S. aureus* incubated with CDs and Cu-CDs2, respectively (fluorescence images were captured under the excitation of 488 and 405 nm for CDs and Cu-CDs2, respectively) (* indicates significant differences, *P < 0.05, **P < 0.01, ***P < 0.001).

The antibacterial effect of CDs against *E. coli* (Gram-negative) and *S. aureus* (Gram-positive) was evaluated using the plate counting method. Figure 4A and B show that the number of colonies decreased obviously on the plates with increasing concentrations of CDs, corresponding to an improvement of antibacterial performance. When the bacteria were treated with 0.1 mg/ml of CDs, there were only a small number of *E. coli* and *S. aureus* on the plates, and the antibacterial rates of CDs against *E. coli* and *S. aureus* reached 99.03% and 99.79%, respectively. Gao *et al.* [12] synthesized CDs by using ampicillin as raw material. When the concentration of CDs were 0.7 mg/l and under visible light irradiation, they showed excellent antibacterial capacity only against *S. aureus*, however, the antibacterial capacity was not obvious in the dark. In contrast, the CDs prepared in this study have the advantages of low cost, short preparation time, excellent antimicrobial capacity against both *S. aureus* and *E. coli*, and easy operation without the use of photodynamic power.

The antimicrobial properties of CDs, Cu-CDs1 and Cu-CDs2 have been also compared. As shown in Figure 4C and D, compared to CDs, the antibacterial properties of Cu-CDs1 increased slightly due to less copper doping, however, Cu-CDs2 had a significantly enhanced antibacterial effect was its antibacterial rates against *E. coli* and *S. aureus* were 99.86% and 100%, respectively. When its concentration was only 0.08 mg/ml, as shown in Figure 4A, even at such concentration, a large number of *E. coli* and *S. aureus* were still present after treatment with the ampicillin sodium antibiotic (AMP), while the number of bacteria decreased substantially after treatment with CDs and Cu-CDs1, and they almost disappeared after treatment with Cu-CDs2. These results showed that the antimicrobial CDs prepared in this study had superior antimicrobial effect compared with traditional antibiotic AMP, and that the antimicrobial properties of CDs could be greatly improved due to copper doping and the antimicrobial effect increased significantly with increasing the copper content. The antibacterial properties of Cu-doped CDs were also reported [40], these Cu-doped CDs (with a copper content of 3.95%) at a concentration of 0.156 mg/ml exhibited significant antibacterial effect against *S. aureus*, however, didn’t work against *E. coli*. In contrast, the Cu-doped CDs prepared in this study have excellent antibacterial performance against both *E. coli* and *S. aureus*.

Next, to explore the mechanism of the antimicrobial effect of CDs. We used scanning electron microscopy (SEM) to observe the morphological changes of bacteria and the disruption of bacterial membranes before and after treatment with antimicrobial CDs. As shown in Figure 4E, the *E. coli* and *S. aureus* without CDs treatment remained typically spherical or rod shape, and the surface was intact and smooth. However, the bacteria were damaged to different degrees after treatment with CDs, Cu-CDs1 and Cu-CDs2. In contrast, Cu-CDs2 caused greater damage to both *E. coli* and *S. aureus*, as reflected by deformation, perforation and collapse of the bacterial membrane, and even the phenomenon of partial bacterial content flowing out. Particularly, form SEM images, it can be observed that the surface deformation of *S. aureus* treated with CDs was less pronounced than *E. coli*. This kind of antimicrobial mechanism of CDs against *S. aureus* might be primarily attributed to the endocytosis of bacterial membranes by CDs, which in turn led to great damage to the DNA or proteins of *S. aureus* and ultimately achieved highly efficient antimicrobial activity.

### Bacterial labeling imaging

We further explored the potential of antimicrobial CDs in bacterial labeling. *E. coli* and *S. aureus* were incubated with CDs and Cu-CDs for a period of time, respectively, and then observed under fluorescence microscopy. As shown in Figure 4F, *E. coli* and *S. aureus* treated without CDs and Cu-CDs2 did not show any fluorescence signal under fluorescence microscope, while the bacteria treated with CDs and Cu-CDs2 showed green and blue fluorescence with wide distribution, respectively. The results showed that CDs and Cu-CDs2 had good labeling imaging effects on both *E. coli* and *S. aureus*, indicating their potential as fluorescent probes for bacterial imaging. Furthermore, the results of bacterial labeling experiments showed that the antimicrobial CDs could bind freely to bacteria and distribute evenly within the bacteria, further verifying the mechanism of endocytosis of CDs by bacterial membranes, which is consistent with the SEM results.

### Characterization of biological properties of carbon dots

#### Antioxidation properties

**Figure 5.**
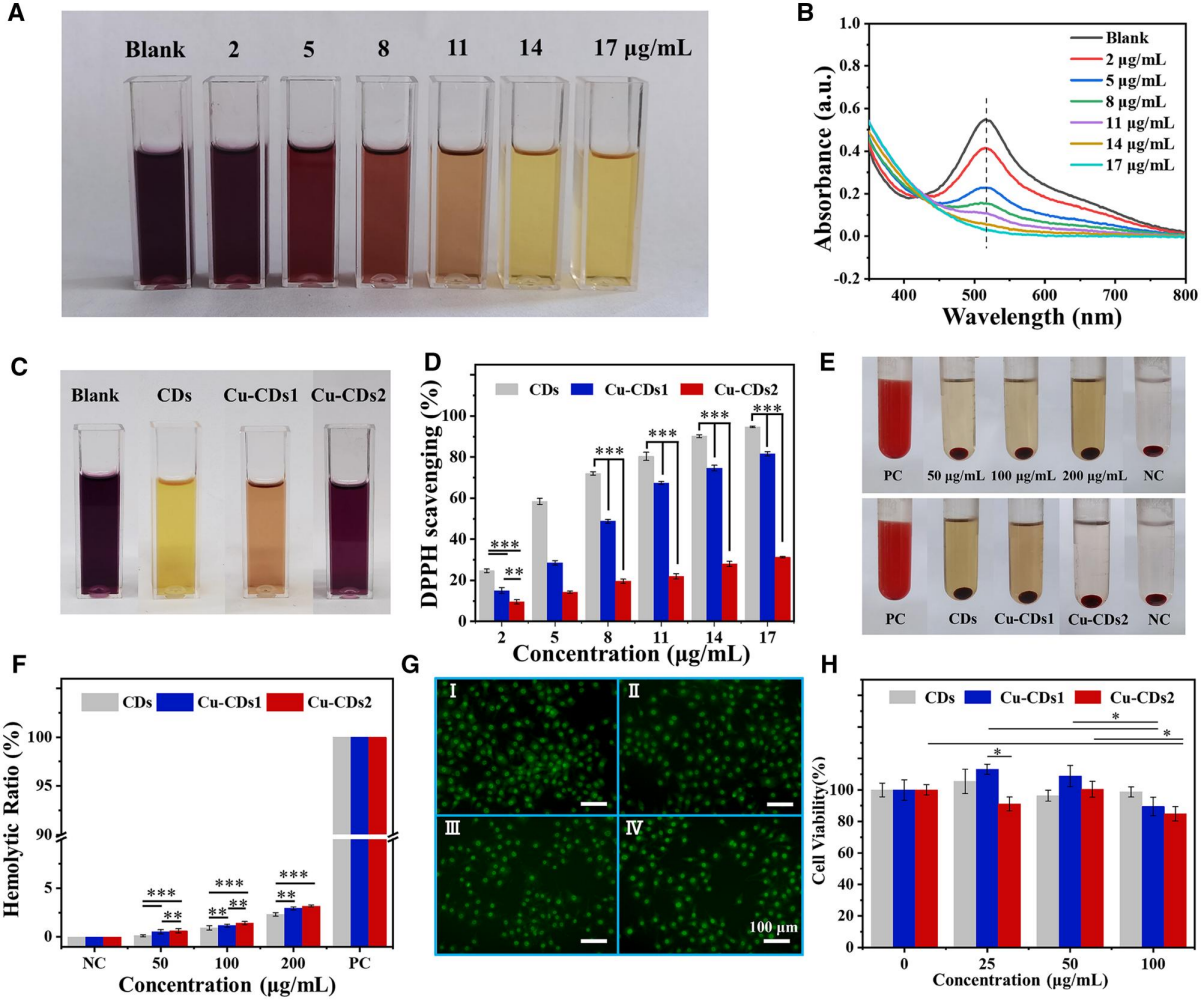
(**A**) Photos and (**B**) UV-vis spectrum of DPPH after co-incubation with different concentrations of CDs. (**C**) Photos of DPPH after co-incubation with CDs, Cu-CDs1 and Cu-CDs2 at a concentration of 200 μg/ml. (**D**) Scavenging rate of DPPH for different concentrations of CDs, Cu-CDs1 and Cu-CDs2. (**E**) Photos of hemolysis for different concentrations of CDs (top) and CDs, Cu-CDs1 and Cu-CDs2 at a concentration of 200 μg/ml (bottom). (**F**) Hemolysis rates for different concentrations of CDs, Cu-CDs1 and Cu-CDs2. (**G**) Fluorescent images of AO/PI stained L929 cells after 3 days of co-culture with CDs (I is the blank group, II is CDs group, III is Cu-CDs1 group and IV is Cu-CDs2 group). (**H**) Cytotoxicity for different concentrations of CDs, Cu-CDs1 and Cu-CDs2 on L929 cells (* indicates significant differences, *P < 0.05, **P < 0.01, ***P < 0.001).

Antioxidant CDs can improve the wound healing process by regulating the excessive production of reactive oxygen species [41–43]. In this study, the antioxidant properties of CDs were evaluated by 1,1-2-phenyl-2-bitter base hydrazyl (DPPH) radical scavenging method which is based on a phenomenon that after scavenging free radicals, the color of DPPH will change from purple to yellow [44, 45]. As shown in Figure 5A, when the concentration of CDs was in the range of 0–17 μg/ml, the color of the solution gradually changed from purple to light yellow as the concentration of CDs increased, corresponding to the weakening of the UV absorption peak at 517 nm in Figure 5B, and the scavenging rate of DPPH radical was subsequently increased. When the concentration of CDs was only 17 μg/ml, the scavenging rate of DPPH reached 94.73% (Figure 5D). This is due to the phenolic hydroxyl groups on the surface of CDs can be oxidized into quinones, which can capture free radicals and then endow CDs with excellent antioxidant properties [46]. We further compared the antioxidant properties of CDs, Cu-CDs1 and Cu-CDs2. With the doping of Cu^2+^ in CDs, the color of the DPPH solution incubated with Cu-CDs1and Cu-CDs2 showed a deepening phenomenon (Figure 5C). This is attributed to the reactions between the phenolic hydroxyl groups of TA and copper ions, which consumed a portion of the phenolic hydroxyl groups and thus diminished the antioxidant performance, but still maintained a certain antioxidant activity, which lays a good foundation for their *in vivo* immunomodulatory capacity.

#### Hemocompatibility

Good hemocompatibility is an important indicator for biomaterials [47, 48]. According to F756-2000 standard from the American Society for Testing and Materials (ASTM), the hemolysis rate of medical devices must be less than 5% [49], for that, we evaluated the hemocompatibility of CDs, Cu-CDs1 and Cu-CDs2. As shown in Figure 5E and F, compared with the positive control (PC), there was no significant hemolysis in the supernatant after treatment with CDs in the concentration range of 0–200 μg/ml. Moreover, after treatment with Cu-CDs1 and Cu-CDs2, the supernatant also did not show significant hemolysis. Even when the concentrations of CDs, Cu-CDs1 and Cu-CDs2 reached 200 μg/ml, their hemolysis rate was still less than 5%, reaching the standard of medical device use. The results showed that in the concentration range of 0–200 μg/ml, CDs, Cu-CDs1 and Cu-CDs2 had good blood compatibility.

#### Cytotoxicity assay

The cytotoxicity of CDs, Cu-CDs1 and Cu-CDs2 on L929 cells was assessed through MTT method. As shown in Figure 5G and H, compared with the blank group, the survival rate of L929 cells decreased slightly with increasing material concentration and doped Cu^2+^ content. When the concentration of CDs, Cu-CDs1 and Cu-CDs2 reached 100 μg/ml, the RGR value remained above 84%. According to the standard, if the RGR value is greater than 75%, it is evaluated as noncytotoxic and can be used as medical materials. In addition, it can be seen that the cell morphology of both the material and the blank group was intact and evenly distributed. The results indicate that CDs, Cu-CDs1 and Cu-CDs2 all exhibit good cytocompatibility, demonstrating that the antimicrobial materials prepared in this study can meet the basic requirements of biomedical materials, which creates the basic conditions for subsequent *in vivo* therapeutic experiments.

### *In vivo* antibacterial and wound healing efficacy

**Figure 6.**
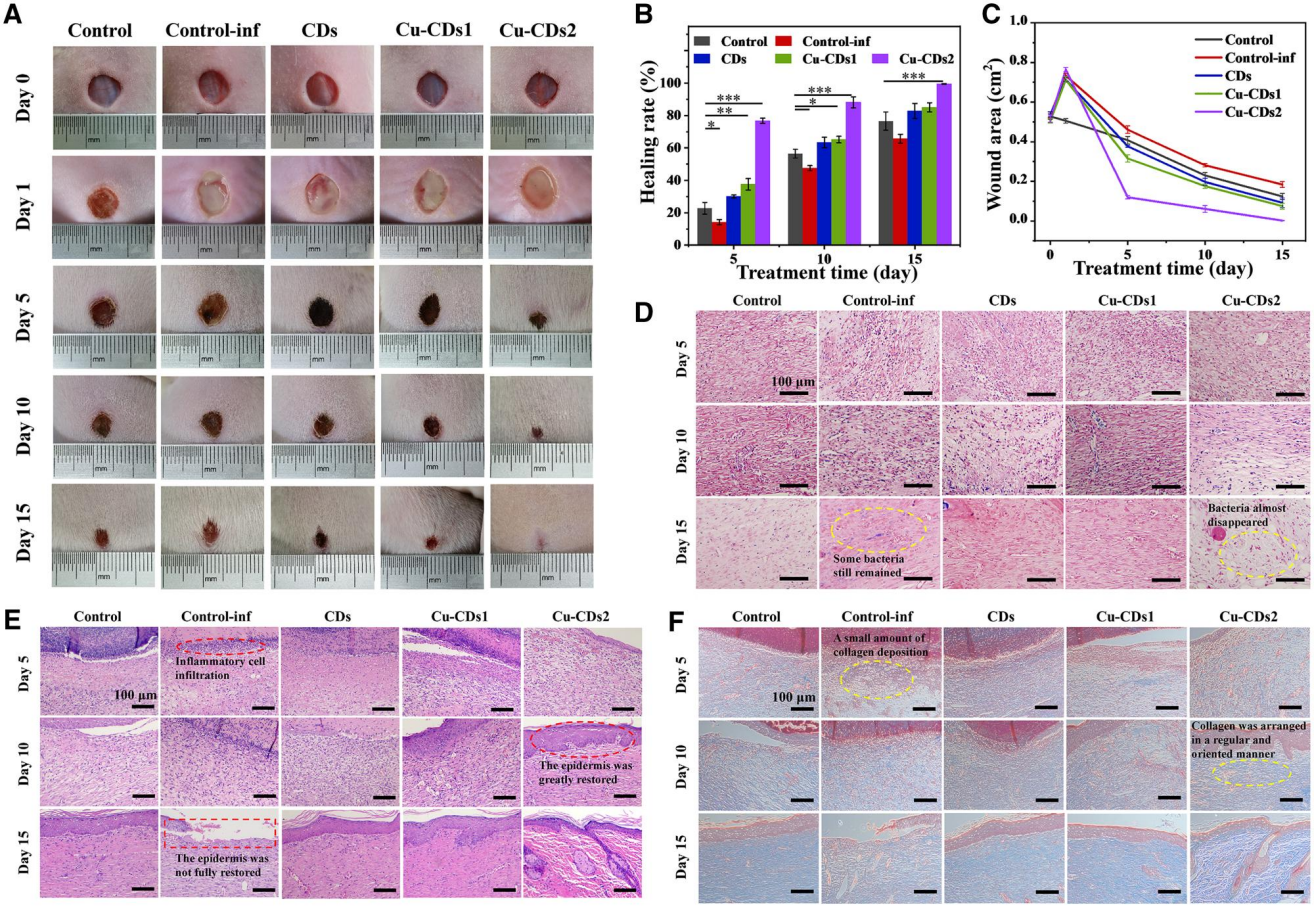
(**A**) Representative photographs of *S. aureus* infected wounds after treatment with different materials on days 0, 1, 5, 10 and 15. (**B**) Wound area and (**C**) wound healing rate after different treatments. (**D**) Gram staining, (**E**) H&E staining and (**F**) Masson staining images of wound tissue in the control group, control-inf group, CDs group, Cu-CDs1 group and Cu-CDs2 group (* indicates significant differences, *P < 0.05, **P < 0.01, ***P < 0.001).

After demonstrating that CDs, Cu-CDs1 and Cu-CDs2 have excellent antibacterial properties, we further investigated their therapeutic ability after bacterial infection on skin wounds of SD rats (Ethical approval number: KS2019052). Firstly, we established a model of total skin defect induced with *S. aureus*, and Figure 6A showed the wound healing process in SD rats, compared with the uninfected control group (Control), the suppuration and inflammation were visible on the wound after modeling (0 day) in each infected group, and the wound area increased compared with the initial size, indicating the successful modeling in this experiment. Subsequently, the wounds treated with CDs, Cu-CDs1 and Cu-CDs2 started to crust and the size of wound was obviously reduced, compared with the infected control group (Control-inf), especially when the wound was treated with Cu-CDs2, its healing was maximally accelerated (Figure 6B). On the 15th day of treatment, both the control group (healing rate of 76.5%) and the control-inf group (healing rate of 65.7%) had some unhealed area. This is because the inflammatory secretions were not controlled in time, the healing speed was slower than that of the material group. CDs have good antimicrobial properties and can effectively inhibit bacteria at the wound site, thus resulting in an increased healing rate (82.9%) compared with the control group, while the wounds treated with Cu-CDs1 showed a higher healing rate (85.1%) on the 15th day of treatment. Compared with the CDs group, the effect of promoting repair for doped CDs is not obvious when they have a lower copper content. However, with increasing Cu^2+^ content, Cu-CDs2 exhibited stronger antibacterial effect and better tissue repair ability. A healing rate of 99.6% was seen on day 15 for Cu-CDs2, showing that Cu-CDs2 significantly accelerated wound healing (Figure 6C). Although photodynamic therapy and antimicrobial gold nanoparticles were also reported to treat bacterially infections and have achieved certain therapeutic effects, the preparation and treatment processes were complex [50]. In contrast, our materials are simpler and less expensive in preparation and our treatment method can achieve good curative effect without relying on any photodynamic power, thus providing a simple and effective way for treatment and tissue repair of infected wounds.

### Histological and biochemical evaluation of skin infected wound healing

*S. aureus* is a Gram-positive bacterium that stains blue-purple in the Gram staining, hence, we can use Gram staining to evaluate bacterial infection of traumatic tissue and the antibacterial effect of materials on *S. aureus*. As seen in Figure 6D, after being infected by *S. aureus*, bacteria could be observed in all groups, among which a lot of bacteria were found in the blank infection group. In addition, because the wound was exposed, some bacteria were also detected in the blank uninfected group. After material treatment, bacteria were effectively suppressed, demonstrating that the as-prepared CDs have excellent *in vivo* antibacterial properties, with Cu CDs2 being the most prominent. To further assess the tissue regeneration of the wound, we performed H&E and Masson staining of the traumatic tissue. As shown in Figure 6E and F, compared with the control and control-inf groups, the materials groups can reduce inflammatory cell infiltration, promote epidermal tissue continuity, and enhance collagen deposition. In particular, after treatment with Cu-CDs2, the epidermis was greatly restored on Day 10, and collagen was arranged in a regular and oriented manner. On Day 15, the infiltration of inflammatory cells has subsided significantly, and the whole skin layer has basically healed and resembles the surrounding normal tissue, and a large amount of collagen deposition and more regenerative attachment formation can be seen, which effectively improved the tissue matrix. The results showed that bacterial invasion greatly hindered wound healing, however, it can be seen that the introduction of the CDs prepared in this study with excellent *in vivo* and *in vitro* antibacterial properties not only promoted wound healing by inhibiting bacteria but also accelerated wound repair and regeneration due to the regulatory effect of Cu^2+^ on promoting vascularization.

**Figure 7.**
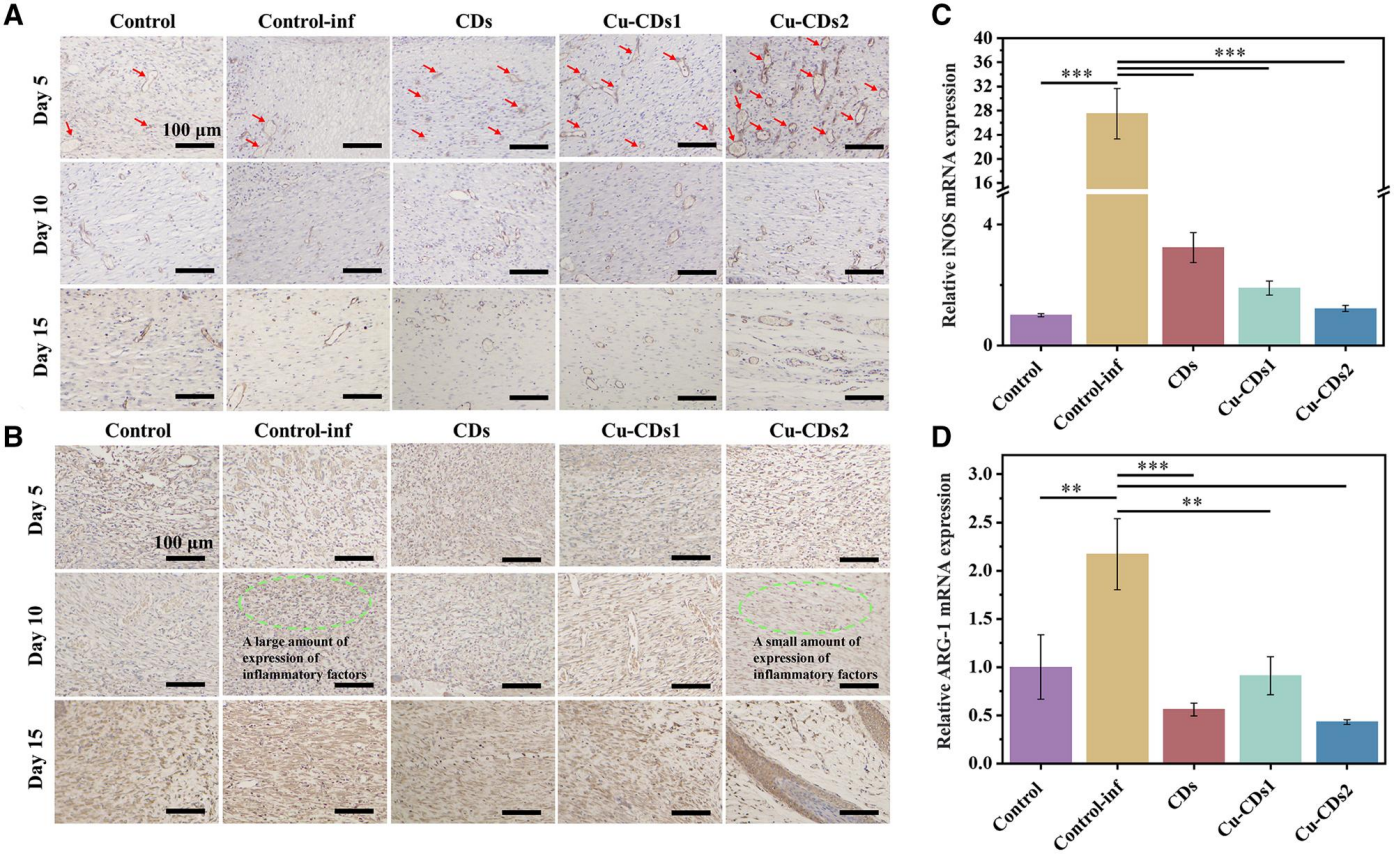
(**A**) TNF-α immunostaining and (**B**) CD31 immunostaining (The arrows point to new blood vessels) images of wound tissue in the control group, control-inf group, CDs group, Cu-CDs1 group and Cu-CDs2 group. (**C**) The relative expression of iNOS mRNA in tissues by real-time quantitative PCR. (**D**) The relative expression of Arginase-1 mRNA in tissues by real-time quantitative PCR (* indicates significant differences, *P < 0.05, **P < 0.01, ***P < 0.001).

To assess inflammation and angiogenesis of traumatic tissue, we performed immunohistochemical staining analysis of TNF-α and CD31. As shown in Figure 7A and B, positive expression of inflammatory factors was observed in all groups. However, the materials group could reduce the expression of inflammatory factors to some extent, which is attributed to the good antioxidant activity of CDs. This endows them with better *in vivo* immunomodulatory properties, resulting in effective inhibition of traumatic inflammatory. Meanwhile, more neovascularization was formed in the wound after treatment with the CDs, especially Cu-CDs2 by virtue of its excellent promoting vascularization effect of Cu^2+^. The results suggest that CDs can play an active role *in vivo* immune modulation and angiogenesis, thereby maximizing the acceleration of wound healing.

There are two phenotypes of macrophages, namely M1 type or M2 type, where M1 type macrophages mainly secrete TNF-α pro-inflammatory factors, which have pro-inflammatory effect and exert host immune function, while M2 macrophages mainly secrete anti-inflammatory factors including IL-4, which have functions of promoting extracellular matrix reconstruction and inflammatory intervention [51]. To quantitatively detect the *in vivo* immune regulatory effect of CDs, we conducted real-time fluorescence quantitative analysis of the expression levels of two phenotypes (M1 or M2) of macrophages. Because they each have respective marker proteins such as iNOS and ARG-1, the ability of immune regulation of CDs can be analyzed by detecting the mRNA expression levels of iNOS and ARG-1. As shown in Figure 7C, when we used the control group as a reference, it can be seen that the mRNA expression level of M1-type marker protein (iNOS) was significantly higher than that in the control-inf group, indicating that M1 macrophages significantly increased and the wounds were still in a high inflammatory phase. However, after treatment with the CDs, Cu-CDs1 and Cu-CDs2, the mRNA expression level of iNOS was significantly reduced, which was due to the excellent antimicrobial properties of the CDs that can inhibit bacterial growth and the good antioxidant activity that can effectively stimulate *in vivo* immune modulation, resulting in a relative reduction of tissue inflammation. In addition, Figure 7D shows the mRNA expression level of M2 type marker protein (ARG-1) was also significantly higher in the control-inf group which was undergoing a high inflammatory phase, indicating that M2 macrophages significantly increased and actively engaged in inflammatory intervention to maintain immune homeostasis. While the mRNA expression levels of ARG-1 in the materials groups were significantly lower than the control and control-inf groups, which might be due to the fact that the materials groups were in the low inflammatory zone or had passed the inflammatory phase, resulting in relatively fewer M2 type macrophages. The results further validated that CDs, Cu-CDs1 and Cu-CDs2 can reduce tissue inflammation and promote wound repair through *in vivo* immune modulation.

To further evaluate the biosafety *in vivo* of the materials, the heart, liver, spleen, lung and kidney of each group of SD rats were removed and analyzed using H&E staining on Day 15 postoperatively. As can be seen in Supplementary Figure S5, there were no obvious histopathological abnormalities such as damage, necrosis or inflammation in any of the groups, indicating that there were no obvious toxic side effects on the organs of SD rats in each treatment group. Therefore, the CDs, Cu-CDs1 and Cu-CDs2 have good biosafety *in vivo*, which makes them possess great potential as an antimicrobial and repair material for skin wounds.

## Discussion

Although there have been sporadic reports on the preparation of antimicrobial CDs using different strategies, there are fewer reports on the hybrid application of CDs in both antibacterial and promoting tissue repair. For example, Miao *et al.* [52] used a method by coupling CDs with antimicrobial materials to obtain antimicrobial CDs, Wang *et al.* [11] and Gao *et al.* [12] synthesized them using materials with stronger antimicrobial properties. The antimicrobial CDs prepared by Yan *et al.* [53] need to be irradiated with blue light to be endowed with antibacterial activity, while the ones prepared by Otis *et al.* [54] showed the antibacterial effect only against Gram-negative bacteria. Additionally, these methods have some drawbacks of cumbersome preparation process, low chemical stability, harsh antibacterial conditions and operation methods and poor broad-spectrum antibacterial activity. Moreover, the above CDs lack the ability to scavenge free radicals induced through inflammatory reactions, thus, it is difficult to achieve optimal tissue repair. In view of this, we designed the CDs with antioxidant and immunomodulatory properties by introducing TA, S and Cu^2+^ with antimicrobial properties. The reasons for choosing S and Cu^2+^ in terms of antibacterial properties are as follows: (i) The sulfur containing structure in the CD can react with some proteins and lipopolysaccharide on the bacterial membrane, causing damage to the integrity and stability of the cell membrane [55]. (ii) The introduction of Cu^2+^ ions into the system can enhance the destruction of the permeability of the cell membrane and cause the imbalance of substances inside and outside the cell, as well as the leakage of cytoplasm, which in turn causes the inactivity of enzymes and interferes with internal metabolic processes such as DNA replication, transcription and repair within the bacteria [56, 57], to achieve an enhanced antibacterial activity.

In addition to antibacterial properties, angiogenesis is another important link in wound healing [58, 59]. The pro-angiogenic function of Cu^2+^ has been reported, which can participate in the regulation of vascular endothelial growth factor, stimulate the proliferation and differentiation of endothelial cells as well as collagen synthesis and deposition, ultimately promoting angiogenesis [60, 61]. Additionally, the use of TA (precursors) in this study can effectively scavenge free radicals and protect wounds from oxidative stress with its antioxidant activity. On the other hand, it can induce *in vivo* immunomodulation to decrease the expression of TNF- and promote the conversion of macrophages from M1 type to M2 type, thereby reducing tissue inflammation. Meanwhile, its immunomodulatory properties can also accelerate the regeneration of new blood vessels in wound tissue [62, 63]. The use of Cu^2+^ and TA created a synergistic pro-angiogenic effect.

Therefore, under the synergistic action of TA, S and Cu^2+^, not only the double efficient antibacterial effect of CDs against Gram-negative and Gram-positive bacteria but also enhanced *in vivo* immunomodulation and angiogenesis could be realized. At the same time, the potential of the antibacterial CDs for bacterial fluorescent labeling provides a great possibility for realizing visual antibacterial and therapeutic. All these will provide huge guarantees for multifunctional CDs in the treatment of infectious wounds.

## Conclusion

TA- and thioacetamide-based antibacterial CDs doped with N, S and Cu^2+^ were successfully synthesized by a simple one-step hydrothermal method. The obtained antibacterial CDs can fluorescently label bacteria and inhibit the proliferation of bacteria. Additionally, the use of tannin (one of precursors used) and ions N, S and Cu allow CDs to achieve stronger functions in antibacterial, immunoregulation and promoting vascularization. Compared with traditional dressing, such antibacterial CDs show better application prospects in treatment of infected trauma. Furthermore, the fluorescence characteristics of such CDs can be expected to realize visual therapy.

## Supplementary Material

rbae105_Supplementary_Data
