# Correction to: Constructing a highly efficient multifunctional carbon quantum dot platform for the treatment of infectious wounds

**DOI:** 10.1093/rb/rbae120

**Published:** 2024-10-18

This is a correction to: Hangzhen Zhang, Jiafan Bai, Xiangli Chen, Linyu Wang, Wenzhen Peng, Yuancong Zhao, Jie Weng, Wei Zhi, Jianxin Wang, Kai Zhang, Xingdong Zhang, Constructing a highly efficient multifunctional carbon quantum dot platform for the treatment of infectious wounds, *Regenerative Biomaterials*, Volume 11, 2024, rbae105, https://doi.org/10.1093/rb/rbae105

In the originally published version of this paper, the panels E and F in Figure 3 were duplicated due to an error in the publication process. This error, for which the publisher apologizes, has now been corrected.

