# Supplementary material for: Constructing a highly efficient multifunctional carbon quantum dot platform for the treatment of infectious wounds
Source: Regen Biomater. 2024 Aug 24;11:rbae105. doi: 10.1093/rb/rbae105 (PMC11377098; doi:10.1093/rb/rbae105)
Supplement: rbae105_Supplementary_Data [file rbae105_supplementary_data.docx]

**Supporting Information**

**Constructing a highly efficient multifunctional carbon quantum dot platform for the treatment of infectious wounds**

*Hangzhen Zhang,^1^ Jiafan Bai,^1^ Xiangli Chen,^1^ Linyu Wang,^1^ Wenzhen Peng,^2^ Yuancong Zhao,^1^ Jie Weng,^1^ Wei Zhi,^1^ Jianxin Wang^1^*, Kai Zhang^3^, Xingdong Zhang^3^*

^1^ Key Laboratory of Advance Technologies of Materials, Ministry of Education, College of Medicine and School of Materials Science and Engineering, Southwest Jiaotong University, Chengdu 610031, China

^2^ Department of Biochemistry and Molecular Biology, College of Basic and Forensic Medicine, Sichuan University, Chengdu 610041, China

^3^ National Engineering Research Center for Biomaterials, College of Biomedical Engineering, Sichuan University, Chengdu 610064, China

*Correspondence address. E-mail: [jwang@swjtu.edu.cn](mailto:jwang@swjtu.edu.cn) (J.W.)

**Materials**

Tannic acid was purchased from the Aladdin Biochemical Technology Co., Ltd. (Shanghai, China). Thioacetamide, copper nitrate trihydrate, and sodium citrate were obtained from Kolon Chemicals Reagent Co., Ltd. (Chengdu, China). Acridine orange (AO), propidium iodide (PI), and thiazolyl blue (MTT) were purchased from Sigma-Aldrich (St. Louis, MO, USA). 1,1-diphenyl-2 bitter hydrazinyl (DPPH) was acquired from Aero Biotechnology Co., Ltd. (Chengdu, China). Animal Total RNA lsolation Kit was purchased from Foregene Co., Ldt. (Chengdu, China).

**Synthesis of CDs, Cu-CDs1 and Cu-CDs2**

Tannic acid (0.3 g), thioacetamide (0.07 g) and copper nitrate trihydrate (0.15 g) were added in deionized water (20 mL) and stirred for 30 minutes to form the precursor solution for the synthesis of CDs. Tannic acid (0.3 g), thioacetamide (0.07 g), and copper nitrate trihydrate (0.8 g) were added in deionized water (20 mL) and stirred for 30 minutes to the precursor solution for the synthesis of Cu-CDs2. Then, the precursor solution was transferred into a 50 mL teflon-lined stainless-steel autoclave in an oven and heated at 200 for 3 h, followed by natural cooling to room temperature. Subsequently, the obtained carbon dots solution was filtered through a 0.22μm filter membrane to remove large particles, and then put into a 500 Da dialysis bag for dialysis using deionized water (RO water) as the dialysate for 24 h. The dialyzed carbon dot solution was filtered through a 0.22μm filter membrane for a second time. Finally, the filtered solution was poured into a petri dish and freeze-dried in a freeze-dryer for 24 h to obtain Cu-CDs2 powder. Cu-CDs1 were prepared using a procedure similar to that described above for Cu-CDs2, for which the amount of copper nitrate required for the preparation of Cu-CDs1 was 0.5 g.

**Characterization Techniques**

The TEM images of carbon dots were obtained using a Tecnai G2 F20 transmission electron microscope. The XRD patterns were performed on a Rigaku Ultma IV X-ray diffractometer. The UV-vis spectra were measured on a UV-2802 UV-vis spectrophotometer. The fluorescence spectra were conducted on a F98 spectrofluorometer. The FTIR spectra were tested on a 5700 Infrared spectrometer. The XPS spectra were analyzed using a Thermo Scientific K-Alpha X-ray photoelectron spectrometer with Al/Ka as the source. The Raman spectra were performed on a XplorRA ONE Raman Microscope excited with 532 nm laser. The zeta potentials were obtained using a Zetasizer Nano ZS90 instrument. Cellular fluorescence images were captured with a IX-51 fluorescence microscope.

**QY Measurements**

Quantum yields (QY) of fluorescent carbon dots were determined by a relative method [1,2]. In this experiment, quinine sulfate and rhodamine 6G were used as standard references, where the quantum yields of quinine sulfate in 0.1 M H_2_SO_4_ were 54%, and rhodamine 6G in anhydrous ethanol were 94%. The equation for calculating the quantum yield is as follows:

$$\text{ϕ}_{\text{x}}\text{=}\text{ϕ}_{\text{r}}\text{×}\frac{A_{r}}{A_{x}}\text{×}\frac{\text{I}_{\text{x}}}{\text{I}_{\text{r}}}\text{×}\frac{\text{η}_{\text{x}}^{\text{2}}}{\text{η}_{\text{r}}^{\text{2}}}\text{ }\text{ }\text{ (1)}$$

Where ϕ is the quantum yield, A is the absorbance at the selected excitation wavelength, I is the fluorescence integrated area, η is the refractive index of the solvent. The subscripts x and r refer to the substance to be measured and the standard reference, respectively. In order to minimize re-absorption effects, the absorbances of the measured standard reference and all samples to be measured were less than 0.05.

**Fluorescence Stability Observation**

To investigate the fluorescence stability of carbon dots, firstly, 400 μL of CDs were added to 400 μL of PBS with different pH values (2-13), and the fluorescence emission spectra were measured under the optimal excitation wavelength, and the fluorescence intensity at the maximum emission wavelength was recorded to investigate the effect of pH on the fluorescence intensity of CDs. Then, 400 μL of CDs were added to 400 μL of sodium chloride solutions with different concentrations (0, 0.25, 0.5, 1, 1.5, 2 mol/L) to investigate the salt tolerance of CDs. Subsequently, 400 μL of CDs were added to 400 μL of hydrogen peroxide solutions with different concentrations (0, 10, 20, 40, 60, 80 and 100 mmol/L) to investigate the antioxidant properties of CDs. Finally, the CDs were continuously irradiated under the excitation light for 1 h. The changes of fluorescence intensity were measured and recorded every ten minutes to investigate the photo-bleaching resistance of CDs.

**In Vitro Antimicrobial Test**

Gram-negative Escherichia coli (ATCC25922) and Gram-positive Staphylococcus aureus (ATCC25923) were used to evaluate the antibacterial properties of the carbon dots by the plate count method. Briefly, 100 μL of bacterial suspension (1 × 10^6^ CFU/mL) was incubated with 100 μL of CDs, Cu-CDs1 and CuCDs2 solutions with different concentrations (final concentrations of 0.02, 0.04, 0.06, 0.08, 0.1 mg/mL), and a blank control group was set up. Then, after incubating at 37 ℃ for 24 h, 100 μL of the co-culture solution was removed and diluted 10 times, and then 20 μL of the dilution solution was removed and applied evenly to the solid medium. After incubation at 37 ℃ for 24 h, the bacterial density was detected by colony counting method to quantify the antibacterial properties of the carbon dots. The equation for calculating the antibacterial rate is as follows:

$$\text{Antibacterial rate=}\frac{\text{M}_{\text{0}}\text{-M}}{\text{M}_{\text{0}}}\text{×100\% }\text{ }\text{(2)}$$

Where M is the average number of colonies in the experimental group and M_0_ is the average number of colonies in the blank group.

The morphologies of bacteria before and after incubation with CDs, Cu-CDs1 and CuCDs2 were observed by SEM. Briefly, E. coli and S. aureus were incubated with CDs, Cu-CDs1 and CuCDs2, respectively. Then, after incubating at 37 ℃ for 4 h, and the bacterial suspension was centrifuged at 6000 rpm/min for 5 min and washed twice with sterile PBS. After removing the supernatant, the bacteria were fixed with glutaraldehyde (2.5%) for 30 min at room temperature. Subsequently, 30%, 50%, 70%, 80%, 90%, and 100% ethanol solutions were used to dehydrate the fixed bacteria step by step for 15 min. Finally, 10 μL of the bacterial suspension was dried naturally on a clean silicon wafer for SEM measurement.

**Bacterial Labeling**

E. coli and S. aureus were incubated with CDs and CuCDs2 in a constant temperature incubator, respectively. Then, after incubating at 37 ℃ for 24 h, the bacterial suspension was centrifuged at 6000 rpm/min for 5 min and washed twice with sterile PBS. After removing the supernatant, the bacteria were re-suspended in sterile PBS. Finally, 10 μL of the bacterial suspension was aspirated on a slide and covered with a coverslip, and placed under a fluorescence microscope for observation.

**Antioxidation Properties**

DPPH was dissolved in absolute ethanol to form a 100 μM solution. Subsequently, it was stored in the dark for later use. 100 μL of carbon dot solution with different concentrations (0, 2, 5, 8, 11, 14, 17 μg/mL) was added to 3 mL of DPPH solution (100 μM), mixed well and reacted in the dark at room temperature for 1 h. The absorbance of the solution after reaction at 517 nm was measured using an UV-Vis spectrophotometer. The equation for calculating the free radical scavenging efficiency of DPPH is as follows:

$$\text{Y=(1-}\frac{\text{A}_{\text{x}}}{\text{A}_{\text{0}}}\text{)×100}\text{ }\text{(3)}$$

A_0_ and A_x_ are the absorbance of the groups without and with carbon dots.

**Hemocompatibility**

Fresh blood from SD rats was centrifuged and the precipitated erythrocytes were washed several times with PBS. And then PBS was added to an EP tube containing pure erythrocyte to form a suspension of erythrocyte with a concentration of 2% (v/v). 1.5 mL of CDs, Cu-CDs1 and CuCDs2 solutions (50, 100, 200 μg/mL) were added to 1.5 mL of erythrocyte suspension, shaken and then incubated for 1 h at 37 ℃ as the experimental group. In the same way, 1.5 mL PBS and 0.2% Triton X-100 solution were added to 1.5 mL of erythrocyte suspension, respectively, as negative and positive controls. Finally, each group of samples was centrifuged at 4000 rpm for 2 min, and 200 μL of supernatant was added in a 96-well plate and tested for absorbance at 540 nm using an enzyme standard. The equation for calculating the hemolysis rate is as follows:

$$\text{Hemolysis rate=}\frac{\text{（}\text{A-}\text{A}_{\text{1}}\text{）}}{\text{A}_{\text{2}}\text{-}\text{A}_{\text{1}}}\text{×100\% }\text{ }\text{(4)}$$

A, A_1_ and A_2_ correspond to the absorbance at 540 nm of experimental group, negative control group and positive control group, respectively.

**Cytotoxicity Assay**

L929 cells were co-cultured with carbon dots to evaluate their cytocompatibility via the MTT method. Briefly, L929 cells were firstly seeded in 24-well plates at a density of 1×10^5^ cells/well, and then incubated in a cell culture incubator at 37 ℃ for 24 h to make the cells grow against the wall. Subsequently，cells were treated with different concentrations (0, 25, 50 and 100 μg/mL) of CDs, Cu-CDs1 and Cu-CDs2, and a blank group was set up. Then, after incubating in the cell culture incubator for 3 days. MTT method was used for quantitative analysis, and UV-Vis spectrophotometer was used to detect the survival of cells at the wavelength of 570 nm. In addition, cells were co-cultured with AO/PI fluorescent dye, and observed and photographed using a fluorescence microscope (Olympus, Japan). The equation for calculating the relative growth rate (RGR) of cells is as follows:

$$\text{RGR=}\frac{\text{A}}{\text{A}_{\text{0}}}\text{×100\% }\text{ }\text{ }\text{ }\text{ (5)}$$

Where A is the average absorbance value of the experimental group and A_0_ is the average absorbance value of the blank group.

**In Vivo Antibacterial and Wound Healing Efficacy**

All animal experiments were approved by Institutional Animal Care and Use Committee at Sichuan University (Chengdu, China, No. KS2019052). The animals used in the experiments were SD rats (50 ± 5 g, 3 weeks old, female), which were purchased from Enswell Biotechnology Co., Ltd. (Chengdu, China) and cultured in an environment (temperature 20-30 ℃, humidity 50-80%) for one week before being used for experimental study. Rats were anesthetized by intraperitoneal injection of chloral hydrate (300 mg/kg) and the back fur was removed, a full-thickness cutaneous defect with a diameter of 8 mm was created with a medical punch on the back of the rats, and then infected with 50 μL of S. aureus (unit concentration 10^8^ CFU/mL) for 24 h [3], and meanwhile a blank uninfected group (Control) was set up. Next day the condition of wound infection was observed to determine whether the modeling was successful. Subsequently, the infected rats were randomly divided into four groups (n=12 per group): blank infected group (Control-inf), CDs group, Cu-CDs1 group and Cu-CDs2 group. Then, 50 μL PBS, CDs, Cu-CDs1 and Cu-CDs2 were applied on the wounds every 24 h from day one, and after natural drying, the rats were put back in the cages. The wounds were administered continuously for 7 days. The wounds were photographed and the wound area was calculated using photoshop software. The equation for calculating the healing rate is as follows:

$$\text{Healing rate=}\frac{\text{A}_{\text{0}}\text{-}\text{A}_{\text{s}}}{\text{A}_{\text{0}}}\text{ }\text{(6)}$$

Where A_0_ is the surgical wound area, A_S_ is the wounds area of each group on days 5, 10, and 15 during the experiment.

The first samples were collected on day 5 after the SD rats were euthanized by dislocating the neck. The wound tissues of three SD rats in each group were preserved in liquid nitrogen, and the wound tissues of three SD rats in each group were fixed in 10% formalin buffer and embedded in paraffin. On days 10 and 15, the wound tissues of three SD rats in each group were removed, respectively, fixed in 10% formalin buffer, and then embedded in paraffin. Finally, the wound tissues were sectioned and stained with H&E, Masson, Gram, TNF-α and CD31 for histological observation. Meanwhile, primers were designed for iNOS mRNA and Arginase-1 mRNA in tissue samples stored in liquid nitrogen, and the relative expression levels of iNOS mRNA and Arginase-1 mRNA in tissue samples were measured using a real-time fluorescence quantitative PCR instrument (QuantStudio ^TM^ 3, ThermoFisher, USA). In addition, to assess the safety of the treatment, the heart, liver, spleen, lung and kidney of the rats in each group were removed on day 15 and stained with H&E.

**Statistical Analysis**

In this study, each experimental part was repeated three times, and the results of each study were expressed as mean ± standard deviation. Students-test was applied to compare between two groups, and one-way analysis of variance (ANOVA) was used for statistical tests for comparison between multiple groups. When * p<0.05, the difference between the two groups was considered statistically significant, ** P < 0.01 and *** p<0.001 indicate very significant differences between the two groups.

**Supporting Figures**


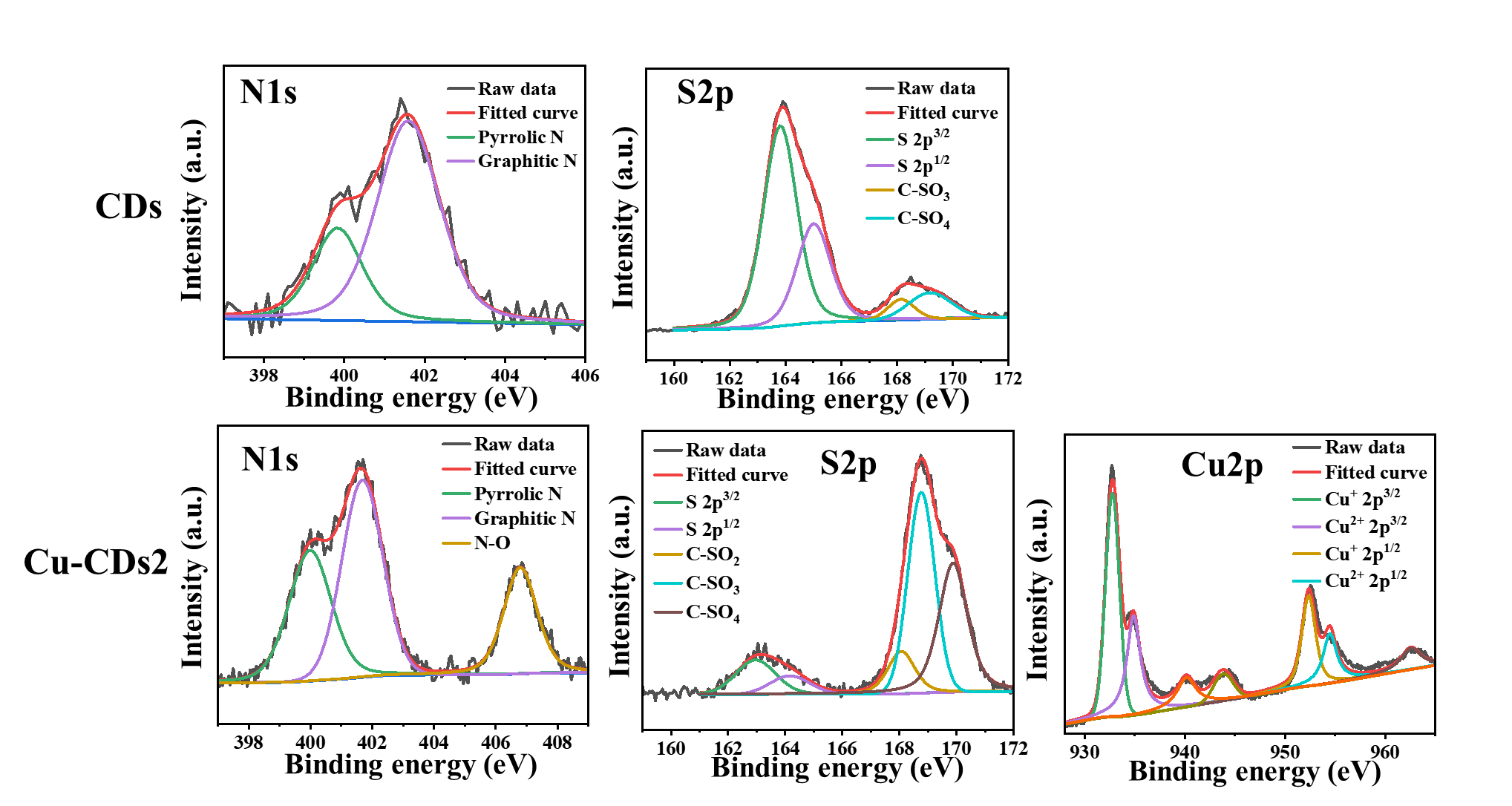


**Figure S1.** High-resolution XPS spectra of N 1s and S 2p of CDs and high-resolution XPS spectra of N 1s, S 2p and Cu 2p of Cu-CDs2.


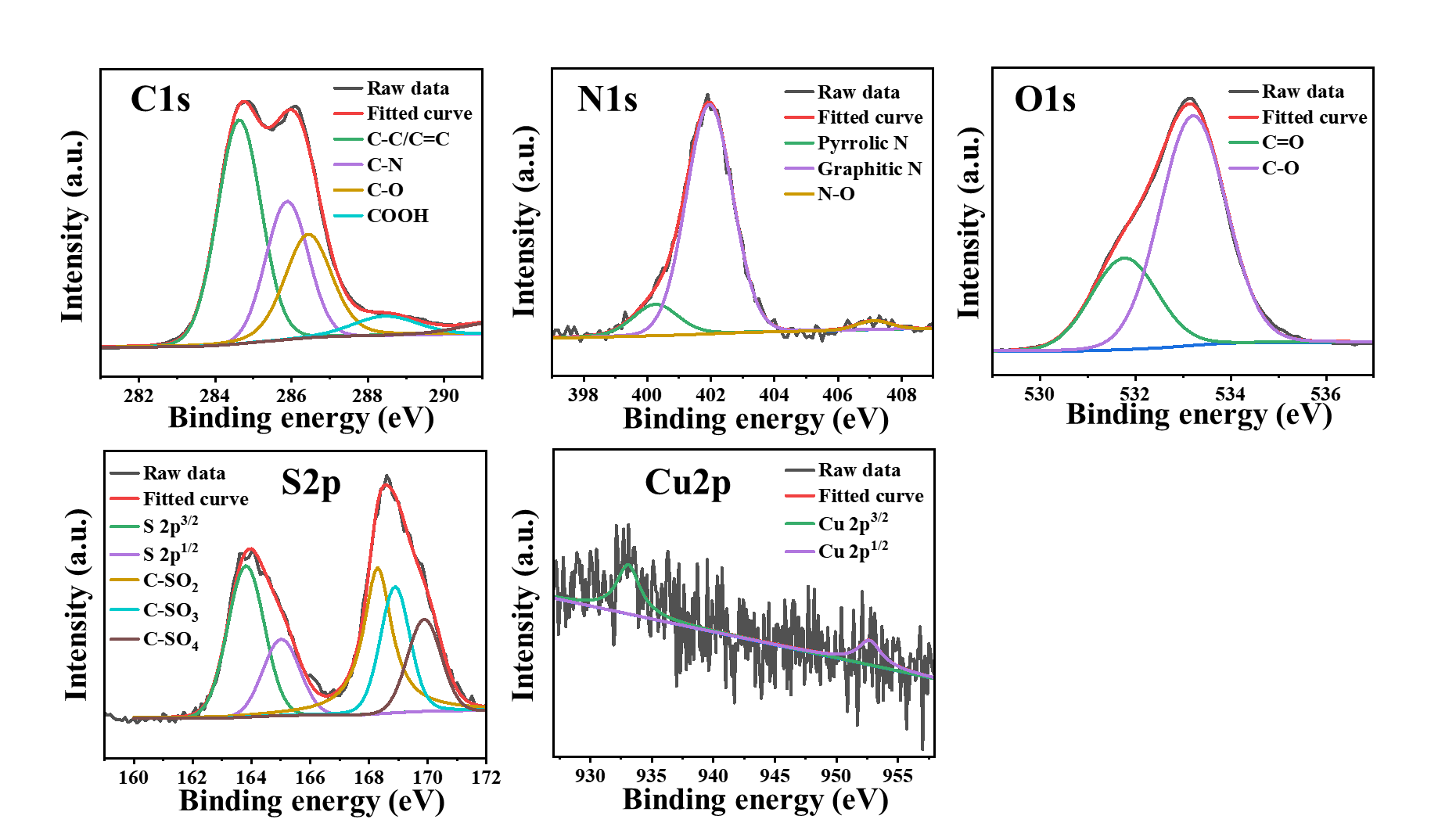


**Figure S2.** High-resolution XPS spectra of C 1s, N 1s, O 1s, S 2p and Cu 2p of Cu-CDs1.


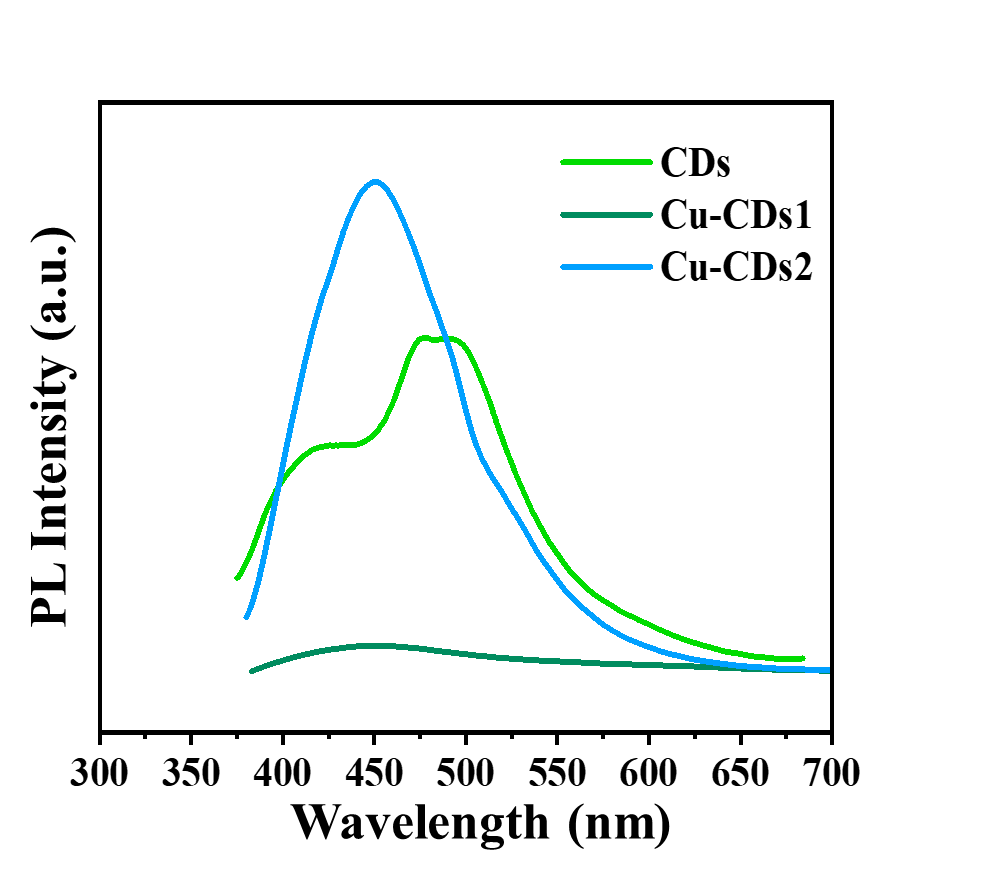


**Figure S3.** CDs, Cu-CDs1 and Cu-CDs2 at 365 nm excitation.


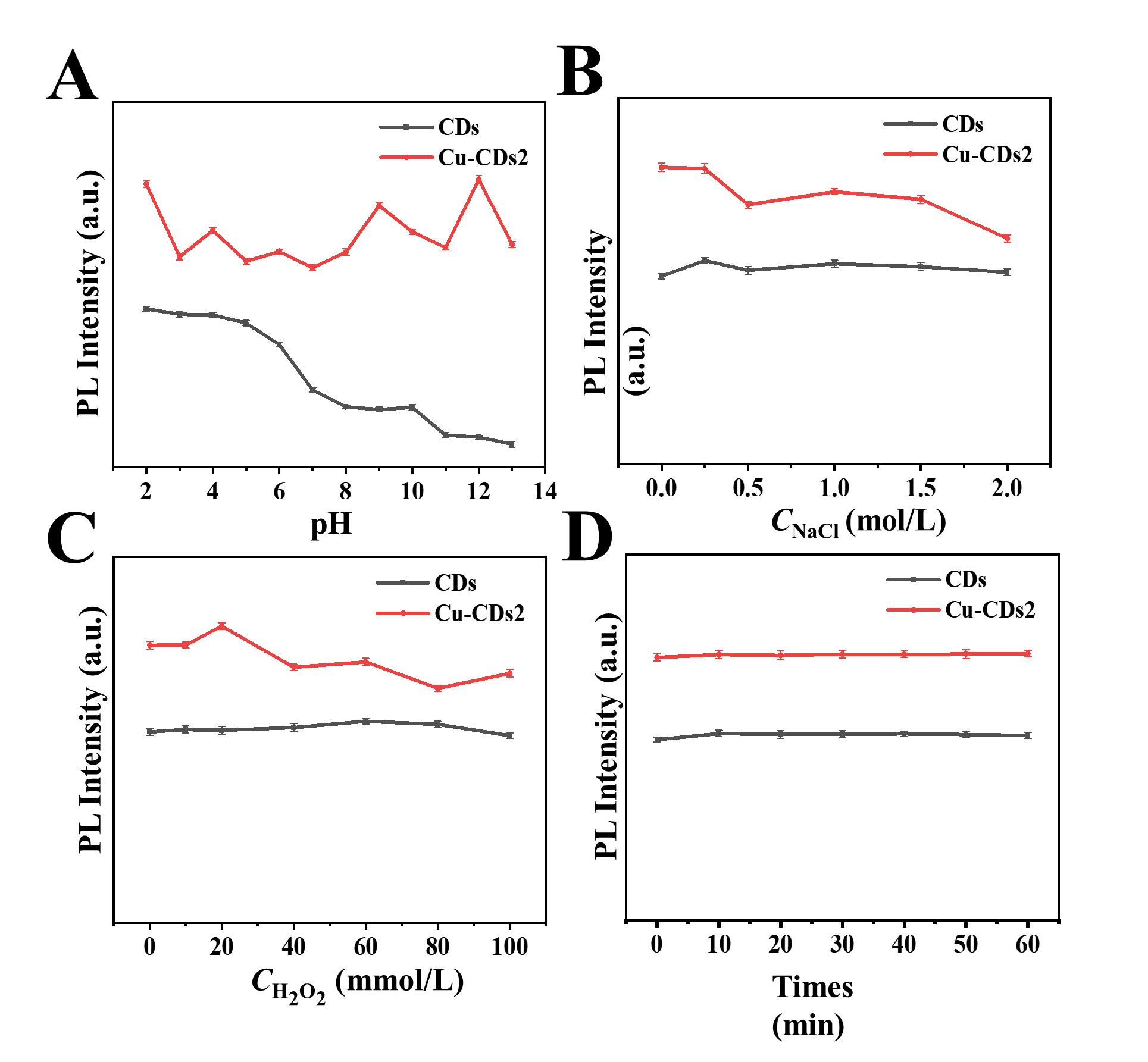


**Figure S4.** The effect of A) pH value, B) concentrations of NaCl and C) H_2_O_2_, and D) laser radiation time on the fluorescence intensity of CDs and Cu-CDs2.


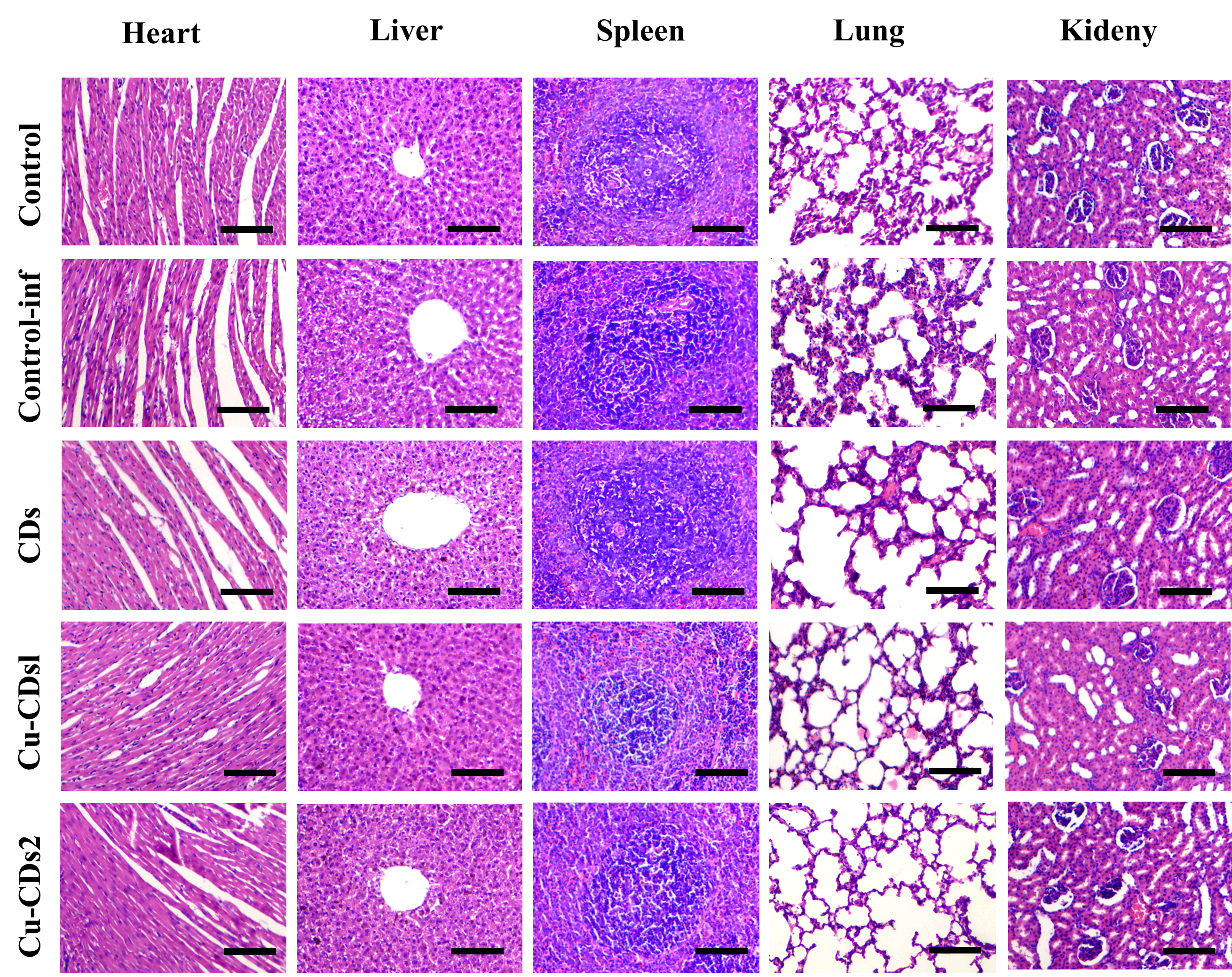


**Figure S5.** H&E staining images of SD rats’ organs in control group, control-inf group, CDs group, Cu-CDs1 group and Cu-CDs2 group. Scale bar = 5 μm.

**Table S1.** The C, N, O, S and Cu element content of CDs, Cu-CDs1 and Cu-CDs2.

| Sample | C (%) | N (%) | O (%) | S (%) | Cu (%) |
| --- | --- | --- | --- | --- | --- |
| CDs | 64.74 | 2.95 | 27.08 | 5.24 | - |
| Cu-CDs1 | 60.39 | 4.13 | 32.11 | 3.24 | 0.13 |
| Cu-CDs2 | 44.7 | 6.15 | 39.26 | 4.24 | 5.65 |

**Table S2.** XPS data analyses of the C1s and O 1s spectra of CDs, Cu-CDs1 and Cu-CDs2.

| Sample | C-C/C=C (%) | C-N (%) | C-O (%) | C=O (%) | COOH (%) | C=O (%) | C-O (%) |
| --- | --- | --- | --- | --- | --- | --- | --- |
| CDs | 48 | 24 | 19 | - | 9 | 35 | 65 |
| Cu-CDs1 | 44 | 26 | 24 | - | 6 | 28 | 72 |
| Cu-CDs2 | 36 | 18 | 19 | 5 | 22 | 67 | 33 |

**References**

[1] Brouwer, Albert M. "Standards for photoluminescence quantum yield measurements in solution (IUPAC Technical Report)*." Pure and Applied Chemistry 2011;83:2213-28.

[2] Jiang, K., Sun, S., Zhang, L., Lu, Y., Wu, A., Cai, C. and Lin, H. Red, Green, and Blue Luminescence by Carbon Dots: Full-Color Emission Tuning and Multicolor Cellular Imaging. Angew. Chem. Int. Ed. 2015;54:5360-3.

[3] Mao C, Xiang Y, Liu X, Cui Z, Yang X, Li Z, Zhu S, Zheng Y, YeungKWK, Wu S. Repeatable Photodynamic Therapy with Triggered Signaling Pathways of Fibroblast Cell Proliferation and Differentiation To Promote Bacteria-Accompanied Wound Healing. ACS Nano 2018;12:1747-59.
